# Egg-associated *Salmonella enterica* serovar Enteritidis: comparative genomics unveils phylogenetic links, virulence potential, and antimicrobial resistance traits

**DOI:** 10.3389/fmicb.2023.1278821

**Published:** 2023-11-10

**Authors:** Ahmed G. Abdelhamid, Ahmed E. Yousef

**Affiliations:** ^1^Department of Food Science and Technology, The Ohio State University, Columbus, OH, United States; ^2^Botany and Microbiology Department, Faculty of Science, Benha University, Benha, Egypt; ^3^Department of Microbiology, The Ohio State University, Columbus, OH, United States

**Keywords:** whole genome sequencing, *Salmonella* Enteritidis, salmonellosis, foodborne disease outbreaks, eggs, egg safety, comparative genomics

## Abstract

*Salmonella enterica* serovar Enteritidis (SE) remains a frequent cause of foodborne illnesses associated with the consumption of contaminated hen eggs. Such a food–pathogen association has been demonstrated epidemiologically, but the molecular basis for this association has not been explored. Comparative genomic analysis was implemented to decipher the phylogenomic characteristics, antimicrobial resistance, and virulence potential of eggs-associated SE. Analyzing 1,002 genomes belonging to 841 sequence types of food-isolated SE strains suggests a high genomic similarity within the egg-related lineage, which is phylogenetically close to SE strains isolated from poultry but is different from those isolated from beef. Core genome- and single nucleotide polymorphism (SNP)-based phylogeny of 74 SE strains of egg origin showcased two distinct sublineages. Time-scaled phylogeny supported the possibility of a common ancestor of egg-related SE lineages. Additionally, genome mining revealed frequent antibiotic resistance due to the presence of *aac(6’)-Iaa* and *mdsAB* encoded on the genomes of egg-associated SE strains. For virulence gene profiling, 103–113 virulence determinants were identified in the egg-associated SE, which were comparable to 112 determinants found in human-associated SE, emphasizing the capacity of egg-associated strains to infect humans and cause diseases. The findings of this study proved the genomic similarity of egg-associated SE strains, and these were closely related to poultry strains. The egg-associated strains also harbor virulence genes equivalent to those found in human-associated SE strains. The analysis provided critical insights into the genetic structure, phylogenomics, dynamics of virulence, and antibiotic resistance of *Salmonella* Enteritidis, circulating in eggs and emphasizing the necessity of implementing anti-*Salmonella* intervention strategies, starting at the production stage of the poultry supply chain.

## 1. Introduction

Non-typhoidal *Salmonella enterica* is estimated to cause 153 million illnesses and 57,000 deaths annually worldwide (Healy and Bruce, 2019). According to the European Food Safety Authority (EFSA) and European Center for Disease Prevention and Control (ECDC), *Salmonella* was involved in 30.7% of foodborne disease outbreaks in 2018, and salmonellosis is the second most reported disease and the second cause of death due to the consumption of contaminated food (EFSA and ECDC, 2019). In the United States, it was estimated that 1 million cases of non-typhoidal salmonellosis occurred in 2013; this infection level accounted for 24% of the economic burden of all foodborne diseases (USDA, 2019). Certain foods are disproportionately linked to salmonellosis outbreaks. Eggs and egg products accounted for 45.6% of salmonellosis outbreaks in the European Union (EFSA and ECDC, 2019). In a recent report by EFSA and ECDC, salmonellosis was the second most reported zoonoses in humans, *Salmonella* Enteritidis remained the most frequent causative agent of foodborne outbreaks, and *Salmonella* serovars in eggs and egg products were of the most concern (EFSA and ECDC, 2022). Similarly, the *Salmonella*–egg combination caused most of the salmonellosis outbreaks in the United States (Dewey-Mattia et al., 2018). In fact, *Salmonella* Enteritidis has been the main serovar associated with human salmonellosis (Ferrari et al., 2019), including those associated with eggs, and the serovar has been associated with disease outbreaks in several countries (Cardoso et al., 2021). The terms “egg” or “eggs,” used in the current study, refer to hen whole shell eggs, egg components such as white and yolk, or raw whole liquid egg.

Despite the obvious *Salmonella* Enteritidis–egg epidemiological association, there is a knowledge gap about the genetic basis for the colonization and survival of *Salmonella* Enteritidis in eggs. Several researchers attempted to determine the underlying genetic mechanisms governing the ability of this serovar to colonize internal egg contents. Previous research showed that non-motile mutants (Δ*fliC*, Δ*fljB*, and Δ*fliH*), compared with the wild-type strain, were impaired in the survival of egg albumin (Cogan et al., 2004; Shah et al., 2012). In addition to motility, genes involved in curli fimbriae production (*agfA*), DNA replication, repair, and recombination (e.g., *yafD*) were significantly important for the survival of the pathogen in egg albumin (Lu et al., 2003; Cogan et al., 2004). In a previous study (Clavijo et al., 2006), screening 2,850 libraries of *Salmonella* Enteritidis mutants revealed the importance of 32 genes, broadly involved in cell wall structure and nucleic acid and amino acid metabolism, for survivability of the serovar in egg albumin. Adaptation of *Salmonella* Enteritidis to egg yolk has been investigated. Passage of *Salmonella* Enteritidis in egg yolk increased intestinal colonization in a colitis mouse model, but the infection dose of the pathogen and the mode of action were not reported in the study (Moreau et al., 2016). In a recent research, the growth of *Salmonella* Enteritidis in egg yolk, in comparison with growth in a synthetic microbiological medium, increased virulence of the pathogen in mice, and a dose of only 280 CFU in yolk was sufficient to kill 50% of the mice (Xu et al., 2022). However, it is still unknown whether *Salmonella* Enteritidis strains isolated from eggs share genomic similarities or can be phylogenomically distinguishable from those isolated from other sources. It is not known whether egg-associated *Salmonella* Enteritidis strains possess important genetic traits (e.g., virulence and antimicrobial resistance) in a similar manner to strains associated with human illnesses. To reduce this knowledge gap, the current study was conducted to provide insight into phylogenetic characteristics, antimicrobial resistance, virulence, and genetic signatures of *Salmonella* Enteritidis associated with eggs.

## 2. Materials and methods

### 2.1. Selection of *Salmonella* strains

To evaluate the distinct global genomic characteristics of *Salmonella* Enteritidis, compared with other serovars isolated from food sources, a dataset consisting of 10,869 strains characterized by 9,235 core-genome multilocus sequence types was extracted from the EnteroBase database. This dataset was subsequently utilized to construct a phylogenetic tree using the rapid neighbor-joining model integrated in EnteroBase, thereby elucidating potential genetic distinctions. Additionally, to further underscore the genetic distinctiveness of *Salmonella* Enteritidis strains originated from eggs compared with those from other food sources, a subset of 1,002 strains represented by 841 core-genome multilocus sequence types was extracted from the EnteroBase database. A minimum spanning tree model, integrated in EnteroBase, was then employed to construct a phylogenetic tree.

For a focused assessment of the phylogenetic attributes, antimicrobial resistance genetic determinants, and virulence traits of *Salmonella* Enteritidis strains associated with eggs, we selected strains from the National Center for Biotechnology Information (NCBI) due to their adequate metadata and the comprehensive genomic sequence information. The selection of *Salmonella* Enteritidis strains was based on a defined set of criteria, ensuring that the strains are representative and suitable for analysis. A total of 94 *Salmonella* Enteritidis strains submitted to NCBI between 2015 and 2021 were used to represent the following two groups, egg-related and non-related groups. The quality of the selected genomes was also performed by assessing the N_50_ and coverage values of the genomes. The ranges of coverage and N_50_ for the selected genomes were 29–301x and 32 kb–4.7 Mb, respectively (Supplementary File 1). The egg-related group included 74 strains isolated from 23 whole raw liquid egg samples, 20 shell eggs, and 31 raw egg yolk samples. These were all the strains that met the selection criteria below and retrieved from the NCBI pathogen isolates database.^1^ The isolates, which were (a) associated with “egg” in their “isolation source,” (b) originated from different single nucleotide polymorphism (SNP) clusters, and (c) not belonging to the same outbreak, were retrieved from NCBI in November 2022. Among the selected egg-related strains, three shell egg isolates (*Salmonella* Enteritidis PT8, PT13, and ODA 99-30581-13) were sequenced in our laboratory at The Ohio State University, Columbus, United States, as described in a previous study (Xu et al., 2021). The second group was a control group (non-egg-related) integrated into the analysis to provide a comparative baseline against egg-related strains. The control group consisted of 19 clinical strains originating from human feces and one strain originating from a farm environment. These strains were selected from geographical regions that were different from those of the egg-related group, and their isolation source metadata explicitly indicated no association with eggs. Detailed information about the investigated strains is presented in Supplementary File 1.

### 2.2. Reference-based single nucleotide polymorphism calling and phylogeny

For SNP calling, genomes of the 94 *Salmonella* Enteritidis strains were uploaded as Fasta files into CSI Phylogeny 4.1.0 tool (available at the Center for Genomic Epidemiology; https://cge.cbs.dtu.dk/services/CSIPhylogeny/; Kaas et al., 2014) and mapped against a reference genome of *Salmonella* Enteritidis strain P125109 (accession number GCA_015240635.1). SNPs were called at default settings (minimum SNP quality of 30, and minimum map quality of 25). CSI phylogeny yields a Newick maximum likelihood phylogenetic tree inferred by FastTree 2.1.7, and the tree was visualized using the interactive Tree Of Life tool (iTOL; https://itol.embl.de/; Letunic and Bork, 2019).

### 2.3. Core-genome-based phylogeny

Pangenomic analysis was performed utilizing the 94 *Salmonella* Enteritidis strains in addition to the reference genome of the P125109 strain using the Roary pipeline (Page et al., 2015). The whole genomes of all strains were annotated using Prokka program, version 1.13 (Seemann, 2014), which yields annotated genomes in the GFF3 format; these genomes were used as an input for Roary that created a multi-FASTA alignment of the core genes with MAFFT (version 7.477; Katoh et al., 2002) at a minimum BLAST percentage identity of 95. The alignment was used to infer a maximum likelihood phylogeny using IQ-TREE (version 1.5.4; Nguyen et al., 2015), and the phylogenetic tree was visualized and annotated with iTOL.

### 2.4. Time-scaled phylogeny

The SNP-based maximum likelihood phylogenetic tree produced by CSI phylogeny tool was time-scaled and rooted by LSD2 (version 1.4.2.2.; To et al., 2016), as described previously (Carroll et al., 2021) using the default settings with modifications as follows: (i) a substation rate of 2.79 × 10^−7^ substitutions/site/year, a rate that corresponds to the estimate determined for *Salmonella* Typhimurium DT104 in a previous study (Leekitcharoenphon et al., 2016); (ii) tip dates corresponding to the collection year for each strain; (iii) genome sequence length of the reference strain *Salmonella* Enteritidis P125109 (4,685,848 bp); and (iv) variances of input branch lengths. The resulting time-scaled phylogenetic tree was visualized and annotated using iTOL.

### 2.5. Identifying antimicrobial resistance and virulence determinants

For AMR determinants, two different genome-mining analyses were applied to *Salmonella* Enteritidis genomes. First, ABRicate (version 1.0.1) in GalaxyTrakr pipeline (Gangiredla et al., 2021) was used to identify the AMR determinants in the 94 *Salmonella* Enteritidis genomes, in addition to the reference genome, *Salmonella* Enteritidis P125109, using the Resfinder database with 80% minimum DNA identity and coverage for each gene. Second, AMRFinderplus (version 3.8.28; Feldgarden et al., 2019) was used to detect AMR and stress response genes using the following settings: AMRFinderplus database (version 2020-09-30.1), *Salmonella* organism option, minimum coverage percentage of 50, and minimum identity threshold of 75. In addition, plasmid replicons, in *Salmonella* Enteritidis genomes, were screened using ABRicate (version 1.0.1) and PlasmidFinder databases with an identity and coverage percentage of 80. For virulence factor determination in the *Salmonella* Enteritidis genomes, ABRicate (version 1.0.1) was implemented against the Virulence Factor Database (VFDB) with a minimum coverage and identity percentage of 80. The presence or absence of AMR and virulence genes were represented as heatmaps using GraphPad Prism software (version 9.4.1; GraphPad Software, San Diego, CA, United States).

## 3. Results

### 3.1. Global genomic diversity of *Salmonella enterica* isolated from food

**Figure 1:**
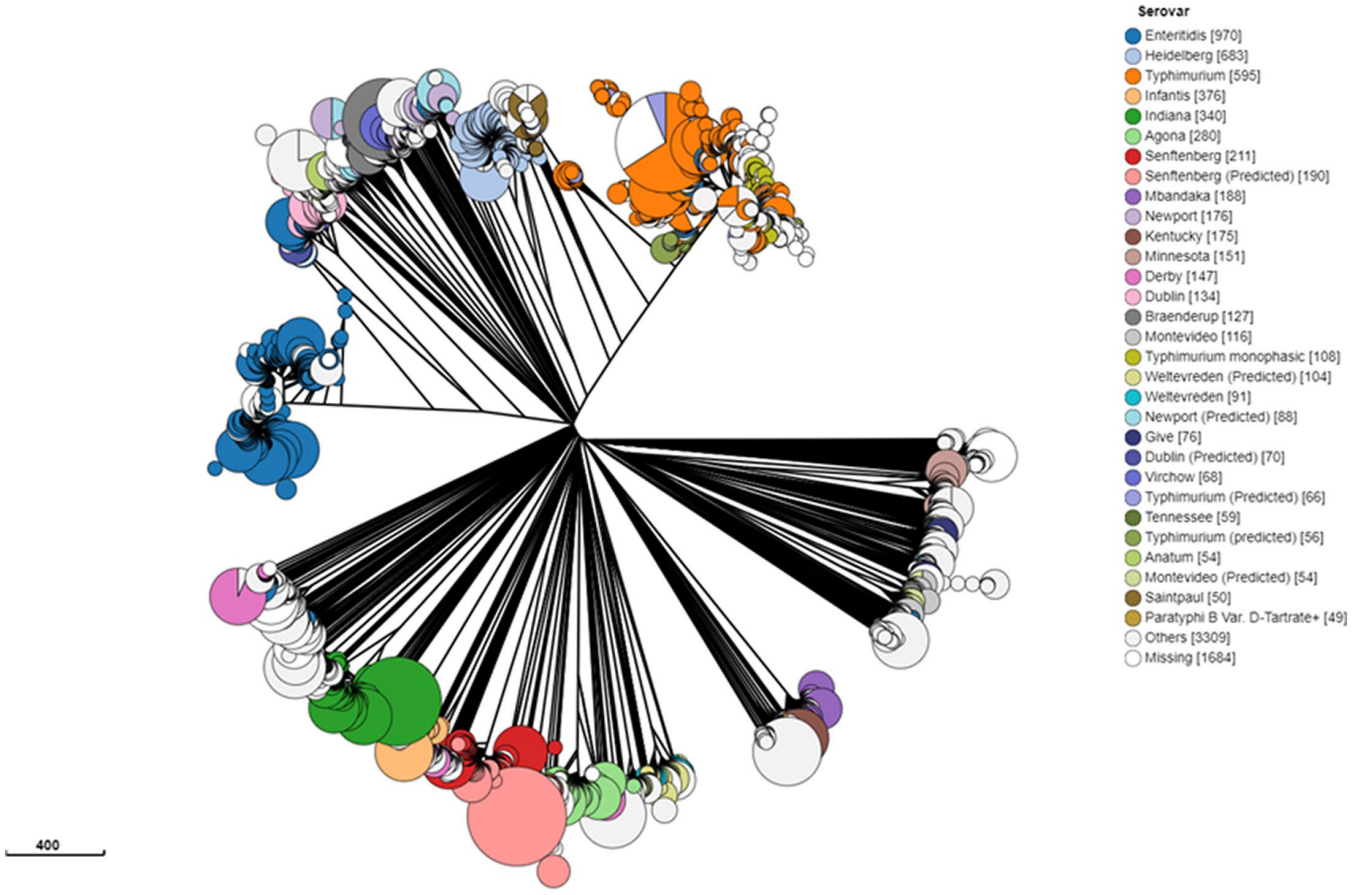
Genomic diversity of *Salmonella enterica* isolated from food worldwide, as determined by a neighbor-joining tree of 10,869 strains of 9,235 core-genome multilocus sequence types, according to EnteroBase. Each node represents a unique sequence type, and the size of each node corresponds to the number of strains within that node. *Salmonella* strains are color-coded by serovar, and the number of isolates is presented between brackets. The scale bar indicates 400 core-genome multilocus sequence-type alleles.

**Figure 2:**
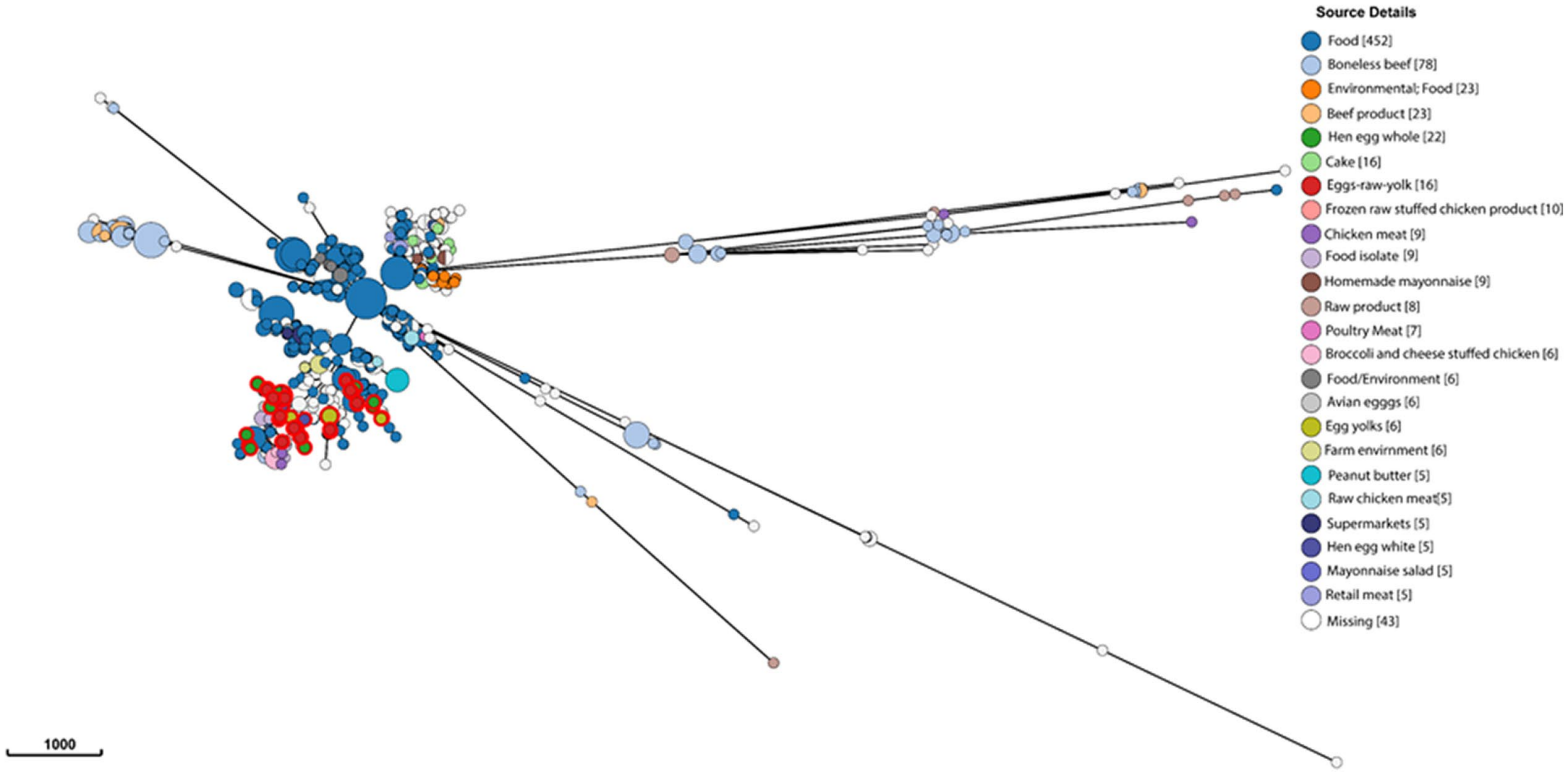
Genomic diversity of *Salmonella* Enteritidis isolated from food, according to the EnteroBase database, and determined by the minimum spanning tree of 1,002 strains of 841 core-genome multilocus sequence types. Each node represents a unique sequence type, and the node size corresponds to the number of *Salmonella* Enteritidis strains within that node. *Salmonella* strains are color-coded by EnteroBase-defined isolation source details (the number of isolates is presented between brackets), and the scale bar indicates 1,000 core-genome multilocus sequence type alleles. Reference to “food” in the legend indicates that the food types were not specified in the EnteroBase.

To gain a global overview of the genetic diversity of *S. enterica* isolated from food, we investigated the population structure and genomic diversity of non-typhoidal *S. enterica* isolated from “food” as a “source niche” according to the EnteroBase database. A neighbor-joining phylogenetic tree (Figure 1) was constructed based on cgMLST and allelic differences; this tree comprised 10,869 strains belonging to 9,235 sequence types (STs) of more than 30 *Salmonella* serovars. The *S. enterica* strains, included in this analysis, were geographically dispersed and originated from more than 30 countries located in Europe, North America, South America, Asia, Africa, and Oceania. Food-associated *S. enterica* strains formed separate clusters when based on the serovar. It was obvious that “Enteritidis,” “Heidelberg,” and “Typhimurium” were the most common serovars in food, each of which formed a distinct lineage in this cgMLST-based phylogenetic tree (Figure 1). The emphasis in this manuscript will be on serovar “Enteritidis” strains from food. By analyzing 1,002 *Salmonella* Enteritidis strains belonging to 841 STs and isolated from more than 20 food sources, the strains were grouped into different lineages (Figure 2), among which one single lineage contained all strains isolated from eggs or egg products (Figure 2, red-colored clusters). This observation indicates relatively small genomic diversity within the egg-related strains. Nevertheless, the egg-related group clustered closely with the poultry-associated strains and distantly from the beef-associated strains (Figure 2).

### 3.2. Phylogenetic relationships within egg-associated *Salmonella* Enteritidis

**Figure 3:**
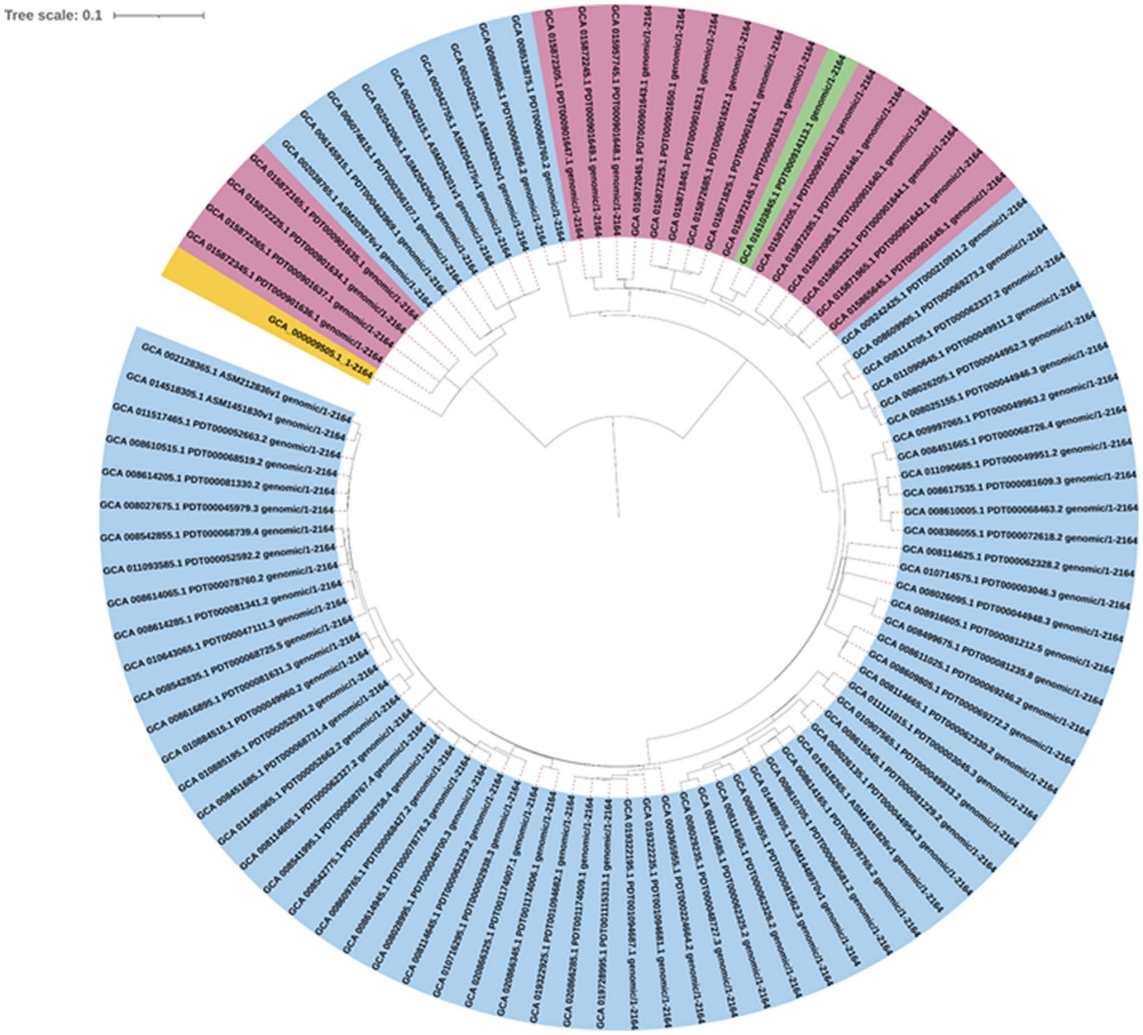
Maximum likelihood phylogeny constructed based on core single nucleotide polymorphism (SNPs; *n* = 2,164) among 94 *Salmonella* Enteritidis genomes. The strains were color-shaded according to their source as follows: eggs (blue), pink (human feces), green (farm environment), and yellow (reference genome of *Salmonella* Enteritidis P125109). Core SNPs were determined by CSI Phylogeny v.4.1.0, whereas the phylogenetic tree was inferred using FastTree v.2.1.7. The scale bar denotes substitutions per site.

**Figure 4:**
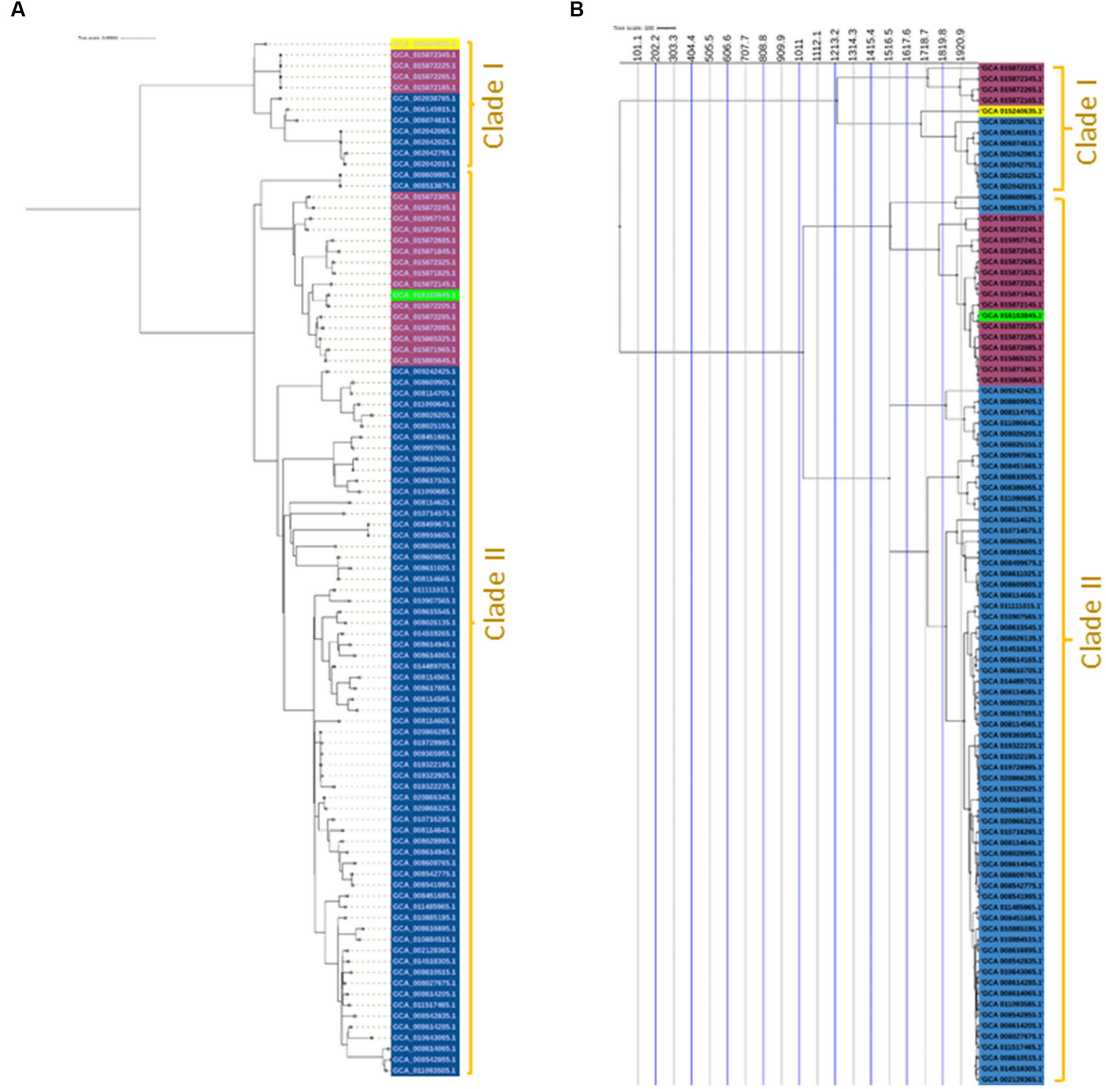
Core-genome and time-scaled phylogeny of *Salmonella* Enteritidis from eggs. **(A)** Maximum likelihood phylogeny of 94 *Salmonella* Enteritidis strains, constructed using the core-genes sequence alignment determined by the Roary pipeline and inferred by IQ-TREE v.1.5.4; **(B)** Time-scaled maximum likelihood phylogeny constructed using core SNPs identified among the 94 *Salmonella* Enteritidis strains; the phylogeny was rooted and scaled using LSD2 with scale bar reported in years. The strains were color-shaded according to their source as follows: eggs (blue), pink (human feces), green (farm environment), and yellow (reference genome of *Salmonella* Enteritidis P125109).

A maximum likelihood phylogenetic tree (Figure 3) was constructed based on 2,164 SNPs detected in the core genome regions of 94 *Salmonella* Enteritidis strains (74 from eggs, 19 from human feces, and one from farm environment for comparison) against *Salmonella* Enteritidis P125109 reference strain. Pairwise comparisons between strains showed a range of SNPs varying from 2 to 542. The SNP-based phylogeny demonstrated that most of the egg-associated strains formed a distinct sublineage from the human-associated strains (Figure 3). These findings were confirmed by constructing a phylogeny (Figure 4A), based on the core genome sequences, in all 94 Enteritidis strains, in addition to the reference P125109 strain. The phylogenetic tree comprised two major clades, and the egg-associated strains formed unique sublineages separated from the human and farm-associated strains (Figure 4A).

A time-scaled phylogeny was built to infer the possible timing of the emergence of the egg-associated *Salmonella* Enteritidis (Figure 4B). The SNP-based time-scaled phylogenetic tree demonstrated the presence of two major clades containing the egg-related strains. A small clade “Clade I” contained a sublineage of seven egg-associated strains that shared a common most recent ancestor dated to approximately 1,920 as determined by the LSD v.2.0.0 tool. The large clade “Clade II” encompassed most egg-associated strains, which formed a distinct sublineage that shared a common most recent ancestor dated approximately to 1,516. Additionally, these findings illustrate the genetic distinctiveness of egg-associated *Salmonella* Enteritidis since most of these strains were clustered in Clade II. Genetic distinction of egg-associated *Salmonella* Enteritidis would become more apparent when more genome sequences from eggs become available in public databases.

### 3.3. Antibiotic resistance and virulence determinants in *Salmonella* Enteritidis from eggs

**Figure 5:**
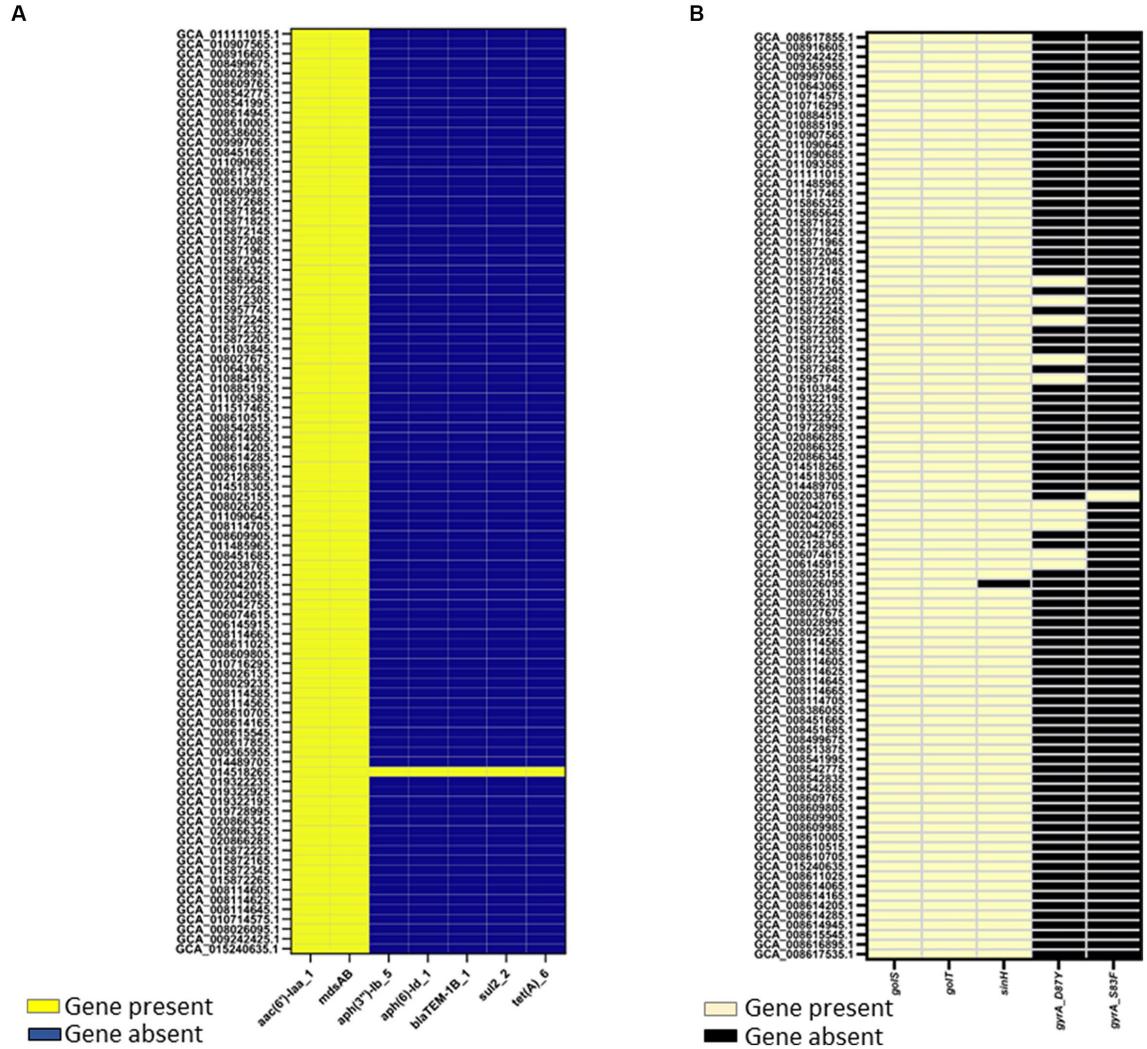
Antimicrobial resistance (AMR) determinants identified in 94 *Salmonella* Enteritidis strains including the 74 strains from eggs. AMR determinants were identified as “present” using ABRicate with the Resfinder database **(A)** at a minimum 80% identity and coverage, or **(B)** AMRFinderplus v.3.8.28 with the AMRFinderplus database at a minimum threshold of 50 and 75% for coverage and identity, respectively. Genes present in *Salmonella* Enteritidis genomes were colored “yellow” in the heatmap illustrations.

Two genome mining approaches, Resfinder and AMRFinderplus, identified AMR determinants in the genomes of egg-associated *Salmonella* Enteritidis while selected human-associated strains (*n* = 19) were used for comparison. AMR results from Resfinder showed that egg-associated *Salmonella* Enteritidis showcased two AMR genes, *aac(6′)-Iaa* and *mdsAB* (Figure 5A), which confer resistance to aminoglycosides and other antibiotics (e.g., chloramphenicol, cephalosporin, and monobactam). These AMR genes were also conserved in the investigated human-related strains. Exceptionally, the egg isolate *Salmonella* Enteritidis PT13 (GCA_014518265.1) harbored genes conferring resistance to aminoglycosides (*aph(3″)-Ib* and *aph(6)-Id*), beta lactams (*blaTEM-1b*), sulfonamide (*sul2*), and tetracycline (*tetA*). With AMRFinderplus analysis, the results (Figure 5B) revealed the presence of a colonization factor (*sinH*), and some stress response genes (*golS* and *golT*) that confer resistance to metal ions (i.e., gold and copper). These genes were similarly found in egg- and human-related strains. Moreover, two *gyrA* mutations (D87Y and S83F) were found in a few *Salmonella* Enteritidis strains isolated from eggs and human feces, and these gene mutations mediate quinolone resistance.

Genomic screening was conducted for plasmid replicons known to harbor antibiotic-resistant genes in *Salmonella* genomes. Out of eight different plasmid replicons identified in *Salmonella* Enteritidis genomes, IncFIB(S)_1 and IncFII(S)_1 were detected in 86 genomes (Supplementary File 2). The plasmid replicons IncN_1, Col156_1, ColRNAI_1, and ColpVC_1 were detected in 2–3 genomes while IncX1_1 and IncX4_2 were found in 10–11 genomes. Most of the egg-associated *Salmonella* Enteritidis genomes harbored IncFIB(S)_1 and IncFII(S)_1, and 9–10 of the genomes harbored IncX1_1 and IncX4_2 plasmid replicons (Supplementary File 2). The presence of such plasmid replicons indicates a potential antibiotic resistance capability of egg-associated *Salmonella* Enteritidis.

**Figure 6:**
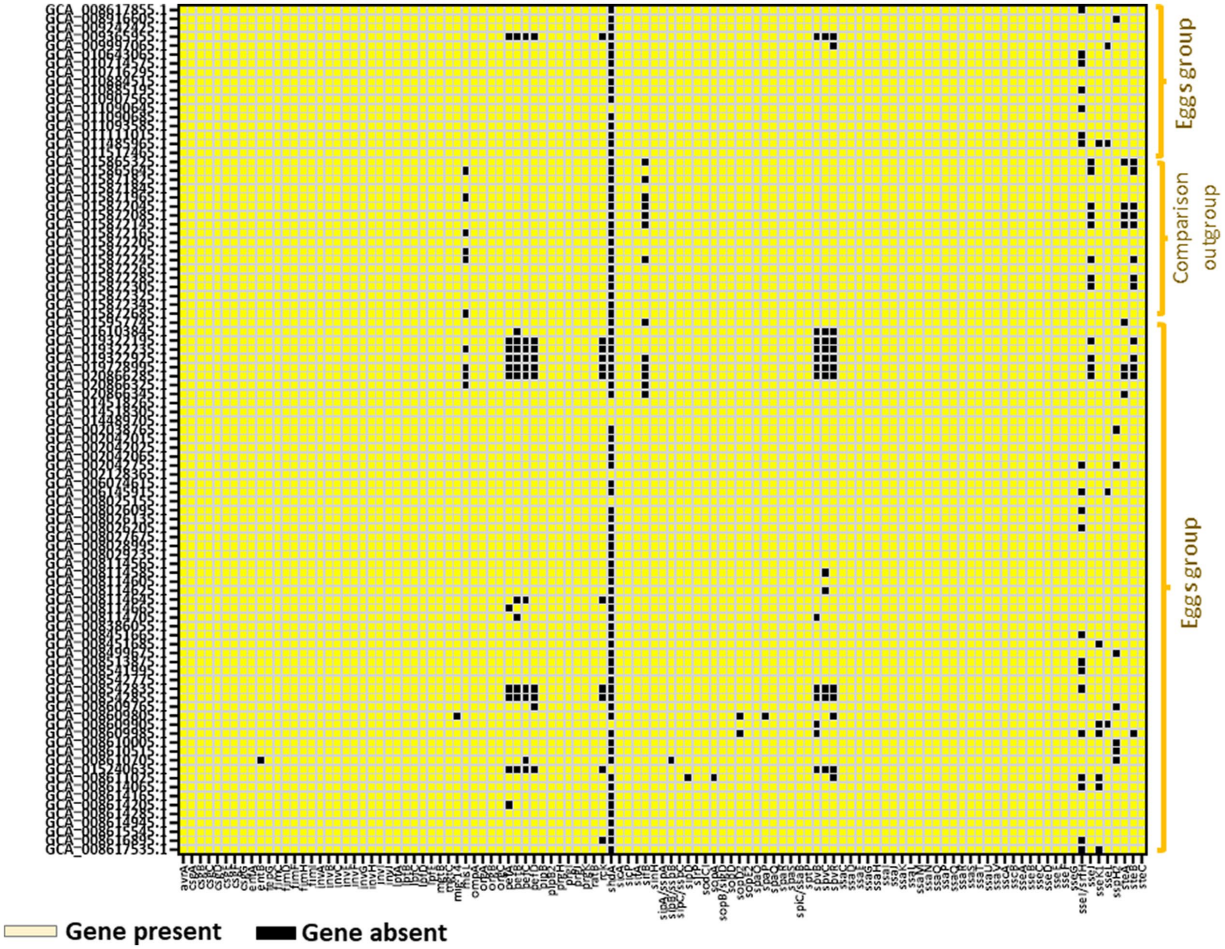
Virulence gene profiling in 94 *Salmonella* Enteritidis strains including the 74 strains from eggs. Up to 113 virulence determinants were identified as “present” using ABRicate with the Virulence Factor database at a minimum of 80% identity and coverage. Virulence genes reported as “present” were colored “yellow” in the heatmap illustration.

Profiling of virulence genes using the ABRicate tool against the virulence factor database revealed that the egg-associated *Salmonella* Enteritidis possessed 103–113 virulence factors in their genomes, mainly encoded on the *Salmonella* pathogenicity islands (SPIs), such as SPI-1, SPI-2, SPI-3, SPI-4, and SPI-5; these SPIs are indispensable for disease onset in the host. Comparably, the human-associated strains harbored 112 virulence genes (Figure 6). These results augment the notion that egg-associated *Salmonella* Enteritidis strains possess putative AMR determinants and the essential virulence factors required for causing human diseases.

### 3.4. Pangenomic analysis highlighted genetic features in *Salmonella* Enteritidis from eggs

The annotated genomes obtained with the Prokka tool were used as an input for the Roary pipeline to infer the pangenome, which is the entire set of genes within the investigated *Salmonella* Enteritidis strains. The pangenome results produced a gene presence/absence matrix by comparing the presence of 6,148 genes with the 94 *Salmonella* Enteritidis genomes (74 for the egg-, 19 for the human-, and one for the farm-associated strains), in addition to the reference *Salmonella* Enteritidis P125109 genome. Our findings (Table 1) showed that two genes (*oadA2* and *oadB2*), involved in sodium ion transport and anaerobic growth, were identified in 30 genomes of the egg-associated strains but were absent in the human-associated group. Additional genes (Table 1) involved in cellular processes, such as DNA-dependent transcription, DNA repair, and conjugal transfer, were identified only in 12–13 of the egg-associated *Salmonella* Enteritidis group but were absent in the human-associated group.

**Table 1:** Proteins and corresponding genes uniquely found in genomes of *Salmonella enterica* serovar Enteritidis from eggs.

Protein (corresponding gene)	Number of genomes^**^ of eggs-associated strains	Biological process
Oxaloacetate decarboxylase alpha chain (*oadA2*)^*^	30	Sodium transport/Biotin binding (Translocase)
Oxaloacetate decarboxylase beta chain (*oadB2*)^*^	30	Sodium transport (Translocase)
Hypothetical proteins	12–14	Unknown
Heme exporter protein C (*ccmC*)	13	Cytochrome complex assembly
Tyrosine recombinase XerC (*xerC*)	13	Cell cycle, cell division, DNA integration, and DNA recombination
Putative transposon Tn552 DNA-invertase bin3 (*bin3*)	12	DNA integration, DNA Recombination
mRNA interferase toxin RelE (*relE*)	12	Toxin-antitoxin system
Transcription antitermination protein RfaH (*rfaH*)	12	Transcription/Transcription regulation
Type IV secretion system protein VirB4 (*virB4*)	12	Conjugal transfer ATPase
Type IV secretion system protein VirB9 (*virB9*)	12	Conjugative transfer
Protein VirD4 (*virD4*)	12	Conjugal transfer
Type IV secretion system protein VirB11 (*virB11*)	12	Conjugal transfer
mRNA interferase HigB (*higB*)	12	Regulation of DNA-dependent transcription, and regulation mRNA stability
Antitoxin HigA (*higA*)	12	Regulation of DNA-dependent transcription
Protein TraL (*traL*)	12	Pilus assembly
Cytochrome c-type biogenesis protein CcmE (*ccmE*)	12	Cytochrome complex assembly
Elongation factor Tu 1 (*tuf1*)	12	Translational elongation, response to antibiotics
DNA topoisomerase 3 (*topB2*)	12	DNA topological change

^*^Anaerobic growth; ^**^Data represent the number of eggs-associated *Salmonella* Enteritidis genomes positive for a given protein that was absent in human-associated strains of the serovar. The term “genome” refers to chromosomal DNA and does not include plasmid DNA.

## 4. Discussion

*Salmonella* Enteritidis is the main *S. enterica* serovar that causes frequent disease outbreaks linked to food, particularly eggs and other poultry products (Rodrigue et al., 1990). The historical dilemma of *Salmonella* Enteritidis–egg association remains unresolved, and understanding the biological mechanisms behind the serovar fitness in such a food matrix is still insufficiently explored. It is unknown whether *Salmonella* Enteritidis strains associated with eggs are genetically identical. Analysis of the global lineages of non-typhoidal *Salmonella* isolated from food (Figure 1) suggests that *Salmonella* Enteritidis is genetically different from the rest of the serovars, and the genomes of the serovar formed a unique cluster. The analysis also showed that *Salmonella* Enteritidis strains from eggs and poultry are closely related genomically (Figure 2), and both are relatively different from the beef-related strains of that serovar. This obvious genetic relatedness emphasizes the importance of the *Salmonella* Enteritidis vertical transmission route, i.e., from breeding chicken to hatching eggs (Gantois et al., 2009). According to a recent study, poultry breeding stocks drove the dispersal of *Salmonella* Enteritidis globally, and that hatching eggs were the key driver for the global spread of this pathogen (Li et al., 2021). The latter study provided evidence that the intercontinental spread of *Salmonella* Enteritidis arose from centralized origins because the global lineages of this pathogen were similar to the strains that were isolated originally from poultry products in the United States and Europe, where the first industrialized poultry production appeared. Moreover, surveys conducted in the United States in 1991 highlighted the presence of *Salmonella* on eggshells from breeder hatcheries (Cox et al., 1991). Together, these findings explain the genomic similarity between *Salmonella* Enteritidis strains from eggs and poultry sources. Intriguingly, *Salmonella* Enteritidis strains from eggs were genetically distinct from those isolated from beef, indicating that beef is an unlikely transmission vehicle of this serovar to eggs.

In the current study, the egg-associated *Salmonella* Enteritidis strains found in the NCBI database (as of November 2022) were included in our analyses. Most of these strains were isolated in the United States; this coincides with the fact that the United States was one of the earliest exporters of chicken breeding stocks to the world (Li et al., 2021). Our SNP- and core-genome-based phylogeny demonstrated that *Salmonella* Enteritidis strains from eggs displayed limited intra-genomic diversity, and these strains were closely related phylogenetically, indicating that they may have a common ancestor. The time-scaled phylogeny constructed in the current study (Figure 4) suggests that the egg-related strains may have naturally evolved from a common ancestor. Comparably, a recent study found that *Salmonella* Enteritidis ST11 lineage from Africa shared a common ancestor dated to 1,551–1,801 (Carroll et al., 2021).

Our findings showcased *Salmonella* Enteritidis strains from eggs to be a reservoir of antibiotic resistance genes, mainly *aac(6′)-Iaa* and *mdsAB*, which encode aminoglycoside acetyl transferase and efflux pump complex, respectively; these genetic traits confer resistance to aminoglycosides and other antibiotics (Alcaine et al., 2007; Song et al., 2014; Nair et al., 2018). The identification of such antibiotic-resistant determinants (Figure 5), in addition to several plasmid replicons (Supplementary File 2), including IncFIB(S)_1, IncFII(S)_1, IncX1_1, and IncX4_1, which have been associated with harboring antibiotic resistance genes (van den Berg et al., 2019; Hull et al., 2022), suggests that the egg-associated *Salmonella* Enteritidis could harbor and possibly disseminate antibiotic resistance genes via plasmid-mediated gene transfer. Antibiotic resistance among *Salmonella* Enteritidis strains from eggs can be explained by the intense use of antibiotics during poultry production (Sapkota et al., 2014). This is consistent with a previous study, which concluded that the prevalence of antibiotic-resistant *Salmonella* was reduced when a conventional farm was converted to organic farming (Sapkota et al., 2014). In addition to that assumption, antibiotic resistance genes can spread among *Salmonella* Enteritidis strains via horizontal transfer of antibiotic-resistant plasmids or chromosomal acquisition of transposons that carry antibiotic-resistant genes (Domingues et al., 2012). The prevalence of *golS* and *golT* in *Salmonella* Enteritidis genomes from eggs highlights the strains’ ability to confer metal ion resistance; the two genes were also implicated in regulating antibiotic resistance efflux pumps (Pontel et al., 2007). These findings alert the egg industry sector to revise the egg/poultry production regimes to reduce the spread of antibiotic resistance among associated pathogens such as *Salmonella* Enteritidis. Our genome (Chromosomal DNA) mining analysis also uncovered a comprehensive virulence machinery in *Salmonella* Enteritidis strains from eggs, which implies the capability of these strains to infect humans and cause diseases.

From a pathogen–food adaptive perspective, the pangenome analysis revealed the abundance of *oadA* and *oadB* and other genes (e.g., *xerC* and *higB*; Table 1) in egg-associated *Salmonella* Enteritidis. These genes enhance the capacity of *Salmonella* to grow anaerobically (similar to the eggshell environment) and in assisting nucleic acid-related processes such as DNA repair in response to damage encountered in environments such as egg white (Woehlke and Dimroth, 1994; Huang et al., 2019). These hypotheses have been overlooked and are now worth further investigation.

## 5. Conclusion

*Salmonella* Enteritidis is a predominant causative agent of egg-associated foodborne disease outbreaks and leads to a multitude of human illnesses and economic losses. This study utilized comparative genomic analysis for an understanding of the phylogenomic links, genomically encoded virulence and antimicrobial resistance traits, and genomic landscape of *Salmonella* Enteritidis strains that were historically associated with disease outbreaks in eggs or egg products. The genetic homology within the egg-associated *Salmonella* Enteritidis indicates a possible common ancestry origin for the circulating strains in egg products, presumably due to the trading of poultry breeding stocks. A whole genome-based comparison gave a glimpse of the virulence aspect of *Salmonella* Enteritidis associated with poultry eggs, and such assessment confirmed the need for advancing the anti-*Salmonella* intervention strategies, starting at the poultry production chain. Further investigations utilizing genome-wide association studies could underpin specific genetic targets characteristics of *Salmonella* Enteritidis associated with eggs.

## Data availability statement

The datasets presented in this study can be found in online repositories. The names of the repository/repositories and accession number(s) can be found in the article/Supplementary material.

## Author contributions

AA: Conceptualization, Data curation, Formal analysis, Investigation, Methodology, Writing – original draft, Writing – review & editing. AY: Conceptualization, Funding acquisition, Project administration, Supervision, Writing – review & editing.

## Footnotes

^1^https://www.ncbi.nlm.nih.gov/pathogens/isolates/
