# Supplementary material for: Egg-associated Salmonella enterica serovar Enteritidis: comparative genomics unveils phylogenetic links, virulence potential, and antimicrobial resistance traits
Source: Front Microbiol. 2023 Nov 10;14:1278821. doi: 10.3389/fmicb.2023.1278821 (PMC10667436; doi:10.3389/fmicb.2023.1278821)
Supplement: Supplementary file 1 [file Presentation_1.PPTX]

## Slide 1
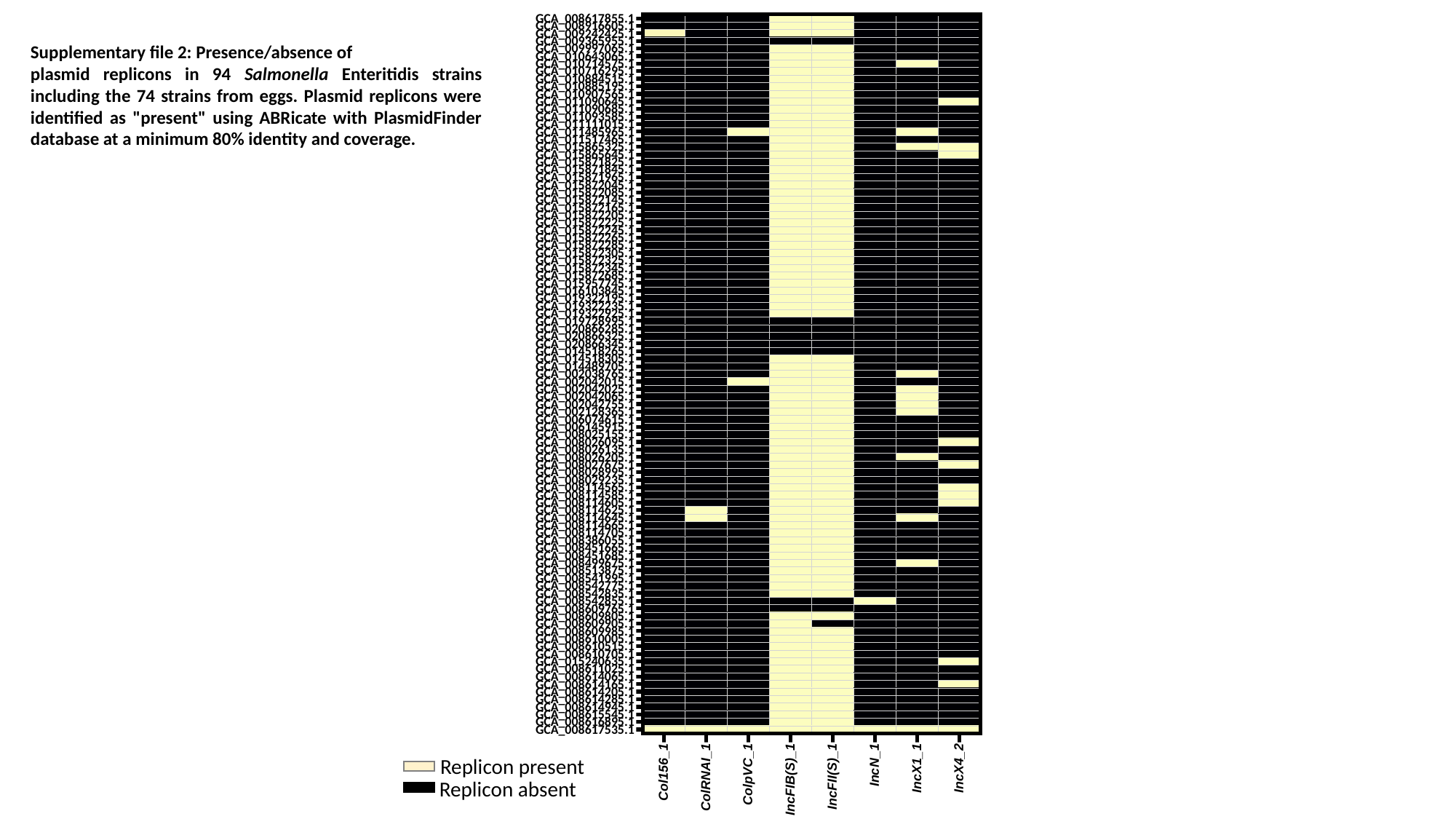

Supplementary file 2: Presence/absence of
plasmid replicons in 94 Salmonella Enteritidis strains including the 74 strains from eggs. Plasmid replicons were identified as "present" using ABRicate with PlasmidFinder database at a minimum 80% identity and coverage.
Replicon present
Replicon absent
